# Metatranscriptomic Analysis of Sub-Acute Ruminal Acidosis in Beef Cattle

**DOI:** 10.3390/ani9050232

**Published:** 2019-05-12

**Authors:** Ibukun Ogunade, Andres Pech-Cervantes, Hank Schweickart

**Affiliations:** 1 College of Agriculture, Communities, and the Environment, Kentucky State University, Frankfort, Kentucky, KY 40601, USA; hank.schweickart@kysu.edu; 2 Department of Animal Sciences, University of Florida, Gainesville, Florida, FL 32611, USA; andrishpech7@ufl.edu

**Keywords:** acidosis, rumen microbiota, metatranscriptomics

## Abstract

**Simple Summary:**

This study evaluated the functional activity of rumen microbiota during sub-acute ruminal acidosis, a metabolic disease of ruminants characterized by low pH caused by feeding highly fermentable carbohydrate feeds. The abundance of rumen bacteria that degrade cellulose (*Fibrobacter succinogenes*, *Ruminococcus albus*, and *R. bicirculans*) were reduced by induced acidotic challenge. Genes mapped to carbohydrate, amino acid, energy, vitamin and co-factor metabolism pathways, and bacterial biofilm formation pathways were enriched in beef cattle challenged with sub-acute acidosis. This study enhances our understanding of the response of rumen microbiota to sub-acute ruminal acidosis by revealing transcriptionally active taxa and metabolic pathways of rumen microbiota.

**Abstract:**

Subacute ruminal acidosis (SARA) is a metabolic disease of ruminants characterized by low pH, with significant impacts on rumen microbial activity, and animal productivity and health. Microbial changes during subacute ruminal acidosis have previously been analyzed using quantitative PCR and 16S rRNA sequencing, which do not reveal the actual activity of the rumen microbial population. Here, we report the functional activity of the rumen microbiota during subacute ruminal acidosis. Eight rumen-cannulated Holstein steers were assigned randomly to acidosis-inducing or control diet. Rumen fluid samples were taken at 0, 3, 6, and 9 h relative to feeding from both treatments on the challenge day. A metatranscriptome library was prepared from RNA extracted from the samples and the sequencing of the metatranscriptome library was performed on Illumina HiSeq4000 following a 2 × 150 bp index run. Cellulolytic ruminal bacteria including *Fibrobacter succinogenes*, *Ruminococcus albus*, and *R. bicirculans* were reduced by an induced acidotic challenge. Up to 68 functional genes were differentially expressed between the two treatments. Genes mapped to carbohydrate, amino acid, energy, vitamin and co-factor metabolism pathways, and bacterial biofilm formation pathways were enriched in beef cattle challenged with sub-acute acidosis. This study reveals transcriptionally active taxa and metabolic pathways of rumen microbiota during induced acidotic challenge.

## 1. Introduction

Subacute ruminal acidosis (SARA) is a metabolic disease of ruminants characterized by low pH, caused by feeding highly fermentable carbohydrate feeds and a consequent accumulation of organic acids [1]. Ruminal pH plays a central role in the normal functioning of the rumen microbiota. Rumen pH below the normal range of 5.8–6.5 for a prolonged duration has significant negative impacts on rumen microbial activity, and animal productivity and health [1,2]. Ruminal acidosis continues to be a big challenge in the dairy and feedlot beef cattle industry and causes financial losses due to inefficient feed utilization and reduced animal performance [3].

Due to the severity of the effects of ruminal acidosis on animal productivity via the disruption of the ruminal microbial environment, several studies have monitored microbial changes during acidosis using quantitative PCR and 16S rRNA sequencing [4,5]. The major effects of ruminal acidosis on rumen microbial population reported by these studies include a reduced number of cellulolytic bacteria, elimination of the protozoal population, and an increased number of amylolytic bacteria. However, microbial changes determined by quantitative PCR and 16S rRNA sequencing do not reflect the function of the microbial population [6]. Shotgun metagenomic sequencing has enhanced our understanding of the rumen microbiota by providing information on the functional potential [7]. However, metatranscriptomics offers the most in-depth information compared to other techniques because of its ability to reveal details about microbial populations that are transcriptionally active [8]. Metatranscriptomics provides an opportunity to investigate functional gene activity and active metabolic pathways. Determining the functional attributes of the microbiome enhances our understanding of their role on animal metabolism and disease [9,10]. Therefore, we applied a metatranscriptomic analysis to reveal a snapshot of the transcriptionally active taxa and the metabolic pathways of rumen microbiota during SARA in beef cattle.

## 2. Materials and Methods

The research protocol was reviewed and approved by the Institutional Animal Care and Use Committee of Kentucky State University (number 19-003).

### 2.1. Animals, Treatments, and Sampling

Eight rumen-cannulated Holstein steers (mean ± SD body weight: 504 ± 45 kg) were assigned randomly to 2 treatments for 18 days (4 steers per treatment). The steers were housed in individual pens and were fed a basal diet composed of 60% red clover/orchard grass hay and 40% concentrate containing corn gluten meal, soy hull, and cracked corn in equal proportions (dry matter basis), ad libitum. The chemical composition of the basal diet is shown in Table S1. Dietary treatments were (1) corn-induced acidotic challenge (CHA; basal diet with induced acidosis) and (2) non-challenge (CON; basal diet without induced acidosis). Sub-acute ruminal acidosis (SARA) was induced as described by Mohammed et al. [4]. Briefly, all the steers had ad libitum access to the basal diet for 16 days. During this time, daily intakes and refusals of experimental diets for each steer were recorded. Samples of the feeds and refusals were collected daily for analysis of DM content to calculate the daily dry matter intake (DMI) of each steer. On day 17, feed was restricted to 50% of the average DMI (calculated from days 1 to 16) for the steers in both CON and CHA groups. On the challenge day (day 18), acidotic challenge was induced in steers in CHA treatment by administering ground corn grain (71.8 ± 1.1% starch), equivalent to 25% of the average DMI (calculated from days 1–16) of each steer directly in the rumen. Immediately after the challenge, the basal diet was offered to all the steers in both CON and CHA treatments for ad libitum consumption.

Representative samples (200 mL) of the ruminal contents for each steer were collected via the cannula by spot sampling from the midpoints along the length and height of the ruminal contents at 0, 3, 6, and 9 h after the morning feeding on the challenge day. At the time of collection, the pH of the samples was measured. The samples collected at 3, 6, and 9 h for each steer were pooled and hand-strained through 4 layers of sterile cheesecloth to separate solid and liquid fractions. The solid and liquid fractions were mixed 1:1 (*w*/*w*) to ensure equal proportions of solid and liquid fractions, and the fractions were stored at −80 °C until metatranscriptomic analysis was done. In addition, subsamples of the liquid fractions were taken and analyzed for volatile fatty acids (VFA) and lactate as described by Ogunade et al. [11]. Briefly, 12 µL of 50% H_2_SO_4_ were added to 12 mL of the liquid portion of ruminal content. The mixture was centrifuged at 11,500× *g* for 20 min. The supernatant was then analyzed for VFA using a Merck Hitachi Elite La-Chrome High-Performance Liquid Chromatograph system (Hitachi L2400, Tokyo, Japan) fitted with a Bio-Rad Aminex HPX-87H column (Bio-Rad Laboratories, Hercules, CA, USA).

### 2.2. Sample Preparation and Sequencing

Approximately 0.25 g of each pooled sample underwent RNA extraction using the RNeasy PowerMicrobiome Kit (Qiagen, Hilden, Germany). The RNeasy PowerMicrobiome kit utilizes a cell lysis protocol that is similar to the PowerSoil DNA isolation kit, which relies on the addition of each sample to a beaded tube in combination with Lysis buffer (Solution PM1/β-mercaptoethanol), and were subsequently vortexed within the Disruptor Genie for 10 min. All RNA extracts were quantified using the Qubit RNA High Sensitivity Kit (Invitrogen, Carlsbad, CA, USA) to confirm complete DNase treatment of the RNA extracts (DNA concentration < 0.05 ng/µL). Subsequently, approximately 100 ng of extracted RNA was subjected to NuGEN Ovation (NuGEN Technologies, San Carlos, CA, USA), double-stranded cDNA synthesis, and metatranscriptome library preparation. The quality of the final library was assessed using a high sensitivity bioanalyzer chip (Agilent, Santa Clara, CA, USA). The metatranscriptome libraries underwent sequencing on the Illumina HiSeq4000 following a 2 × 150 bp index run at the UC Davis Genome Center.

### 2.3. Sequence Quality Assessment and Filtering

Raw read quality was assessed using the FastQC program to obtain average Q scores across the read length of all sequences. The program Trimmomatic was utilized to quality-filter the raw sequence data [12]. A sliding window filtration was utilized to cut reads at a 4-base average Q score of 20 or lower. Reads trimmed below 100 bp were discarded [12]. Post filtration, reads were subjected to the host, *Bos tarus*, cDNA subtraction using *Kneaddata* (version 0.5.4-https://bitbucket.org/biobakery/kneaddata) in sensitive mode. Finally, quality-trimmed non-host reads were merged using BBMap [13].

### 2.4. Taxonomic and Functional Gene Profiling

Functional gene annotation and the quantification of filtered sequence data was conducted using HUMAnN2 with an e-value threshold of 1e^−5^ [14]. Filtered reads were first mapped against the Uniref90 functional gene database, which were subsequently regrouped as KEGG orthologies (KO). Reads per kilobase counts underwent counts per million (CPM) normalization to account for differences in sequencing depth among samples, resulting in a final read per kilobase per million (RPKM) data matrix. For bacterial taxonomy annotation, filtered reads were annotated using the KRAKEN2 software package, using an elevated confidence score threshold of 0.1 [15]. Bacteria comprising less than 0.05% abundance were filtered and discarded from the dataset. Taxonomy counts underwent relative abundance normalization prior to all downstream visualization.

### 2.5. Enumeration of Selected Ruminal Bacteria Using Quantitative Reverse Transcription PCR

Quantitative reverse transcription PCR (qRT-PCR) was further performed to validate the abundance data of *Ruminococcus albus*, *Streptococcus bovis*, and *Megasphaera elsdenii*. qRT-PCR reactions were carried out on a Rototorgene Q (Qiagen) using previously published primer sets that target the 3 bacterial species [16,17]. Positive controls were utilized to confirm the acuity of the utilized primers. Quantification of DNA was conducted to identify and exclude any DNA contamination.

### 2.6. Data and Statistical Analysis

Alpha diversity rarefaction curves were created within the QIIME 1.9.1 package using an unnormalized bacterial count table [18]. Multiple rarefactions were performed on all samples using a minimum depth of 23,000 sequences to a maximum depth of 230,000 sequences, with a step size of 23,000 sequences for 20 iterations. Rarefactions were then collated and compared between CON and CHA treatments using the observed species and Heip’s evenness diversity metrics. Alpha diversity comparisons were conducted using a two-sample *t*-test and non-parametric Monte Carlo permutations (*N* = 999) within QIIME 1.9.1. Partial least squares discriminant analysis (PLS-DA) was performed on the CPM normalized KEGG metatranscriptomic data and bacterial relative abundance data using the mixOmics R package [19]. The PLS-DA model was trained using a 10-fold cross validation, and this model underwent 150 iterations. CPM normalized KEGG gene counts were averaged within CON and CHA treatments for Pathview (version 3.6) plotting [20].

Relative abundances of taxonomic profiles and RPKM-normalized functional gene counts were formatted as described by Segata et al. [21]. Linear discriminant analysis (LDA) effect size (LEfSe) comparisons were made between CON and CHA groups. An alpha level of 0.05 was used for both the Kruskal–Wallis and pairwise Wilcoxon tests. LDA scores greater than 2.0 were displayed for taxonomy and 1.0 for functional (KEGG) genes. Resulting taxonomic and functional gene biomarkers between CON and CHA treatments were identified, and then plotted in LEfSe as differential feature abundance plots. Additionally, significantly differential functional genes were incorporated into a *Phyloseq* object and were plotted as a two-way clustered heatmap within R studio using the *pheatmap* package [22,23]. Additionally, functional genes enriched in both treatments were mapped to the KEGG reference metabolism pathway using iPATH3 [24].

## 3. Results and Discussion

### 3.1. Rumen pH and Fermentation

**Figure 1:**
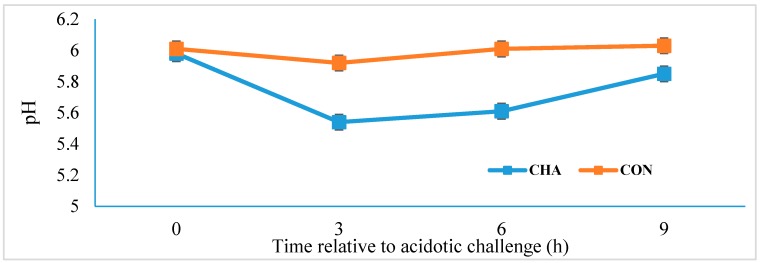
Rumen pH of beef cattle during acidosis challenge. CHA = corn-induced acidotic challenge, CON = control (no challenge).

The mean rumen pH was lower (*p* = 0.04; SE = 0.09) in CHA (5.75) compared to CON treatment (5.99). In fact, the pH value of CHA treatment was below 5.8 for 6 h after the acidotic challenge (Figure 1).

The total VFA and the propionate concentrations were greater in CHA compared to in CON treatment, but lactate concentration was unaffected by dietary treatment (Table 1). This indicates that SARA was successfully induced in this study. Sub-acute ruminal acidosis is characterized by low rumen pH caused by high VFA concentration, without lactate accumulation, as a consequence of feeding highly fermentable grain diets [1]. Though the pH threshold for acute ruminal acidosis varies among studies, the number of hours that ruminal pH stays in the range of 5.2 and 5.8 for a prolonged period is often used to characterize SARA [4].

### 3.2. Rumen Metatranscriptomic Analysis

A range of 5.5 million–19.1 million sequences per sample were retained after the quality filtration and read merging. Over 2300 unique KEGG genes were identified across the entire dataset (Table S2), with a range of 591–938 unique expressed genes per sample (Table S3). About 133 unique transcriptionally active bacterial species were identified across the entire dataset (Table S4).

**Figure 2:**
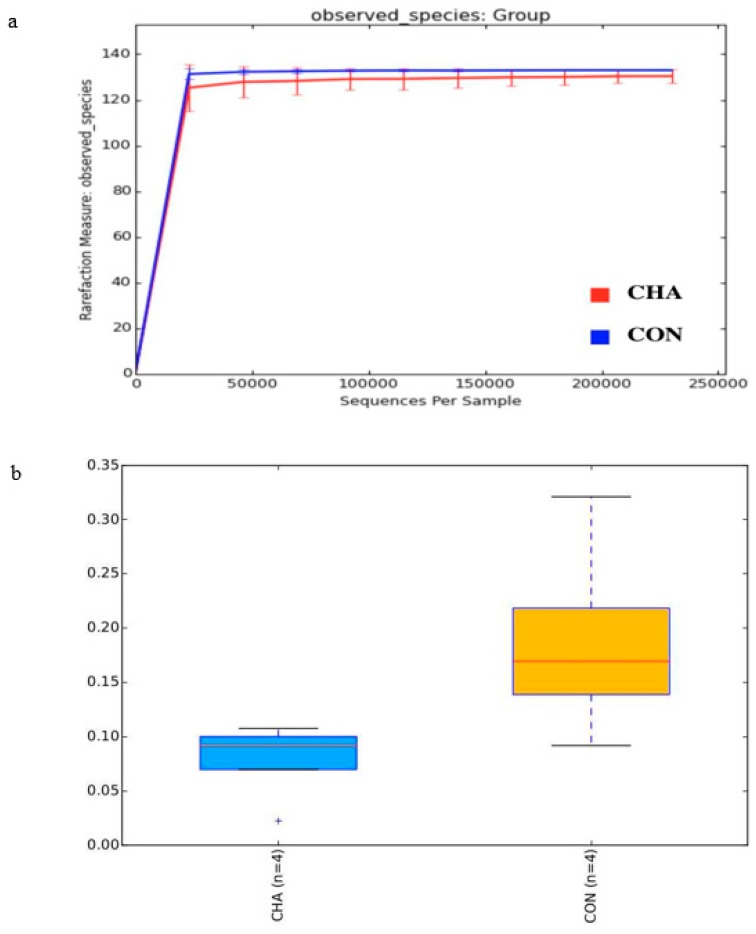
(**a**) Species richness (rarefaction curve) and (**b**) species evenness (Heip’s estimators) of the transcriptionally active microbial community in beef cattle during the acidosis challenge. CHA = corn-induced acidotic challenge, CON = control (no challenge).

Alpha diversity analysis revealed a decreased (*p* = 0.09) species richness within CHA (130.4) in comparison to CON (133.0) (Figure 2a). The species evenness (Heip’s estimator) of the transcriptionally active community was also reduced (*p* = 0.05) by CHA treatment in comparison to CON (Figure 2b). The reduction in species diversity observed in this study is a reflection of lower pH because low ruminal pH reduces the number of bacterial species, particularly the pH-sensitive species. Consequently, there is increased dominance (reduced evenness) of the low pH-tolerant microbial population [25,26]. This result agrees with Petri et al. [27] who reported reduced rumen bacterial richness and diversity in beef cattle fed a high-grain diet.

**Figure 3:**
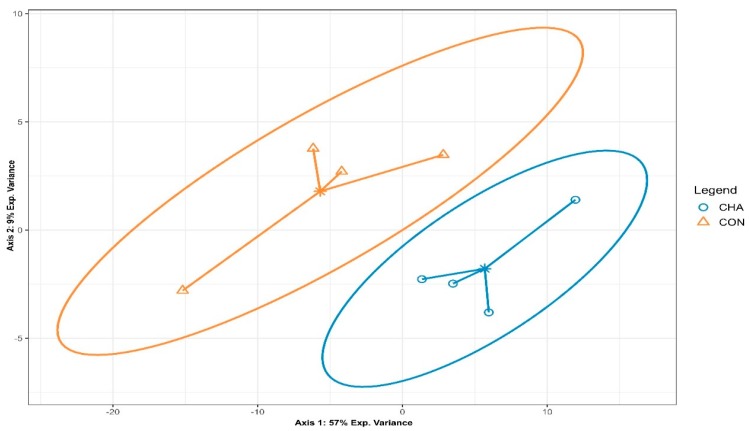
Partial least squares discriminant analysis of the microbial community composition in beef cattle during the acidosis challenge. CHA = corn-induced acidotic challenge, CON = control (no challenge).

Partial least squares discriminant analysis revealed the differential microbial community composition between CHA and CON groups, when considering the relative abundance of identified bacterial species (Figure 3).

**Figure 4:**
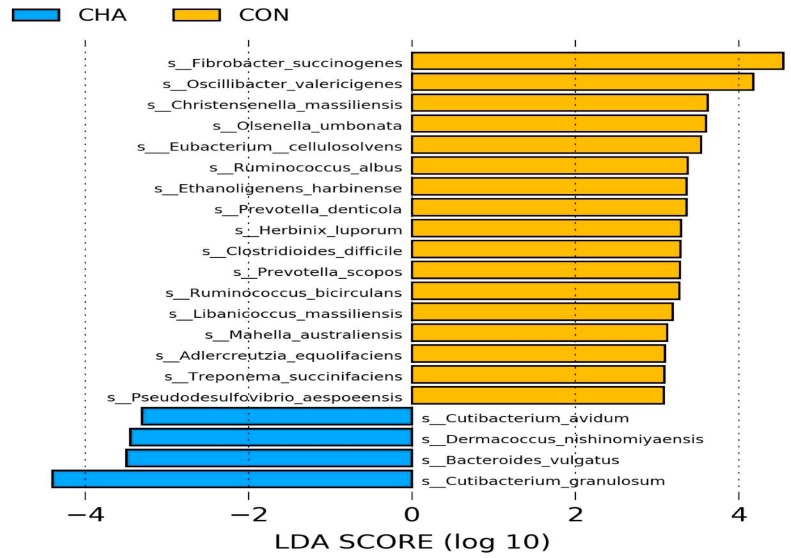
Linear discriminant analysis effect size (LEfSe) of rumen microbiota in beef cattle during the acidosis challenge. CHA = corn-induced acidotic challenge, CON = control (no challenge). The plot indicates the differentially abundant transcriptomic active taxa found by ranking according to their effect size (≥ 2.0) at the species level. The taxa enriched in beef cattle fed the control diet (CON) are indicated with a positive score (yellow), and taxa enriched in the corn-induced acidotic challenge treatment (CHA) have a negative score (blue). Only taxa meeting the significant threshold of 2.0 are shown.

Linear discriminant analysis effect size analysis (LEfSe) revealed 21 differential (LDA > 2.0, *p* < 0.05) taxa between CON and CHA treatments, considering the relative abundance of the identified transcriptionally active taxa (Figure 4). Four bacterial species were enriched whereas 17 bacterial species were reduced by CHA treatment. Among those reduced by CHA treatment were bacterial species from the most studied genera of rumen fibrolytic strains such as *Ruminococcus albus*, *Ruminococcus bicirculans*, and *Fibrobacter succinogenes.* Other fibrolytic bacteria, including *Herbinix luporum*, *Treponema succinifaciens*, and *Eubacterium cellulosolvens* were also reduced by CHA treatment.

Fibrolytic bacteria are sensitive to pH [1] and do not grow at pH ≤ 5.8, a threshold pH value for SARA [28]. Reduced activity and abundance of these bacteria causes reduced ruminal degradation of fiber, an important component in ruminant diets, both as an energy source and for optimum rumen function [29]. Consequently, SARA causes feed intake depression and reduced diet digestibility [30].

*Prevotella* spp. can degrade starch and grow better at low pH [17]. In fact, *Prevotella* spp. have been evaluated as probiotics to control SARA [31]. In this study, *P. denticola* and *P. scopos* were reduced by SARA. To the best of our knowledge, our study is the first to report the response of *P. denticola* and *P. scopos* to SARA challenge. The response of genus *Prevotella* to SARA induction varies across different studies [32]. Numerous studies have reported no effect or an increased abundance of *Prevotella* spp. during SARA [33,34]. However, all of these studies have either focused on *P. bryantii* and *P. brevis* using quantitative RT-PCR [32,35] or limited their results to the genus level using 16S rRNA gene sequencing [5]. The use of metatranscriptomics in this study provides the opportunity to reveal less dominant but active microbial communities. The functions of *P. denticola* and *P. scopos* in the rumen have not been described; both species are common isolates of the human oral cavity. A strain of *Prevotella scopos* is reported to be very active at pH 6–7, with a marginal growth at pH 5 [36].

In this study, the relative abundance of two species of *Cutibacterium* (*C. avidum and C. granulosum*) were increased by corn-induced acidotic challenge. Species of *Cutibacterium*, formerly called *Propionibacterium*, are most active at pH 5–6 [37] and produce propionic acid as an end-product of sugar fermentation [38]. This probably explains the increased propionate concentration observed in this study. In fact, the results of the LEfSe analysis revealed *C. granulosum* (LDA = 4.40; *p* = 0.02), along with *F. succinogenes* (LDA = 4.55; *p* = 0.02), can serve as potential taxonomic biomarkers of SARA in ruminants.

Strains of *Bacteroides vulgatus* and *Demacoccus nishinomiyaesis* have been reported to utilize starch efficiently and can grow at a pH as low as 5.0 [39,40]. This explains their increased abundance in the CHA group due to the increased availability of corn-starch. However, both bacteria are considered human pathogens [40,41], and their prevalence in animals fed high-grain diets warrants further investigation.

Results of the qRT-PCR analysis agree with those of metatranscriptomic data. qRT-PCR analysis revealed that the abundance (copy number per µL × 10^6^) of *R. albus* was reduced (*p* = 0.01) by CHA treatment (5.0 vs. 11.6, SE = 1.43). The abundance (copy number per µL × 10^6^) of *S. bovis*, a lactate-producing bacterial species, was unaffected (1.9 vs. 2.4, SE = 0.38) while that of *M. elsdenii*, a major lactate-utilizing bacterial species, was below the detection limit. The lack of treatment effect on the abundance of *S. bovis* explains the lack of effect on lactate concentration, while the absence of *M. elsdenii* might be attributed to the minimal lactate concentrations observed in this study.

**Figure 5:**
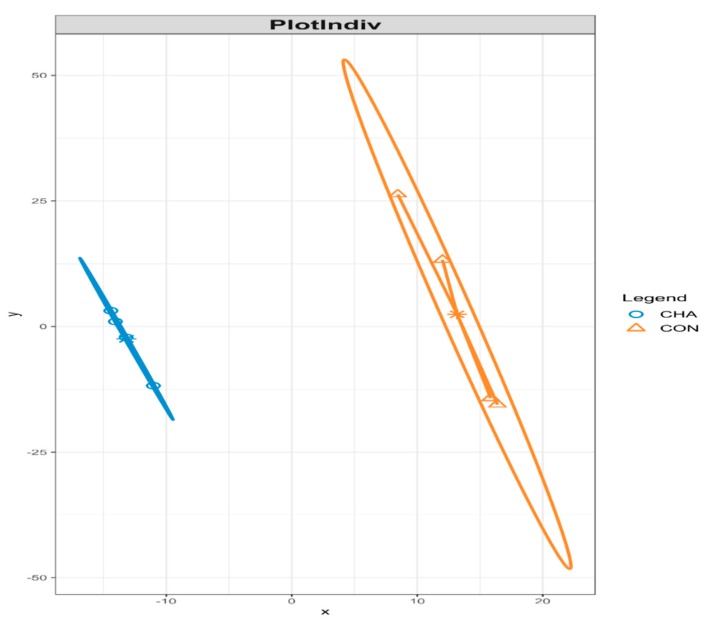
Partial least squares discriminant analysis of functional gene expression profiles in beef cattle during acidosis challenge. CHA = corn-induced acidotic challenge, CON = control (no challenge).

**Figure 6:**
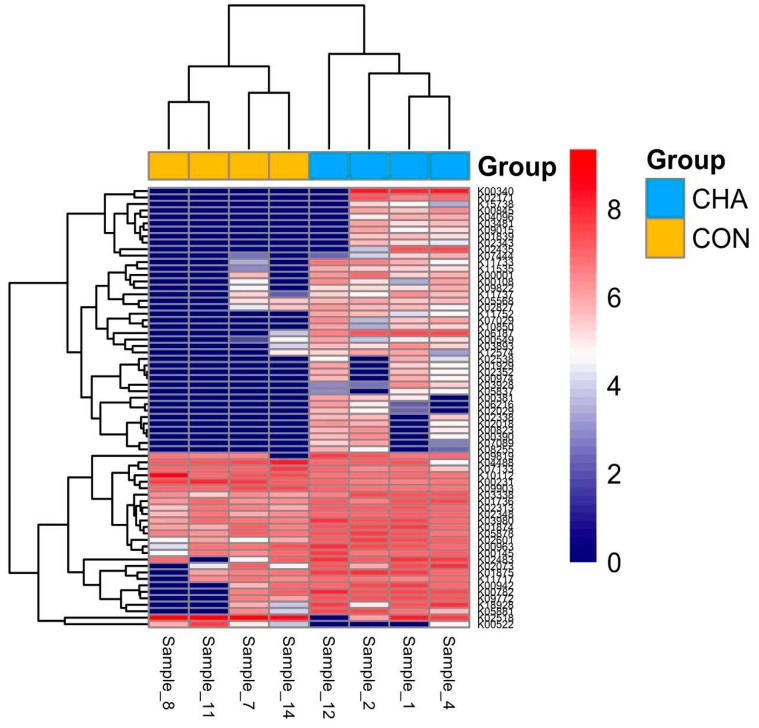
Heatmap analysis showing distinct gene expression profiles in beef cattle during acidosis challenge. CHA = corn-induced acidotic challenge, CON = control (no challenge). CHA = samples 12, 2, 1, and 4, CON = samples 8, 11, 7, and 14. Each row represents one functional gene set.

Partial least squares discriminant analysis revealed differential functional gene expression profiles between CHA and CON treatment groups when considering the RPKM-normalized KEGG annotations identified within each sample (Figure 5). Linear discriminant analysis effect size analysis revealed 68 functional genes that were differentially (LDA > 1.0, *p* < 0.05) expressed between CON and CHA treatments (Table S5). Seven functional genes had decreased expression while 61 genes were over-expressed in CHA treatment compared with CON. The heatmap analysis reveals the distinct gene expression profiles within CHA and CON groups (Figure 6).

When the differential genes were mapped to KEGG using iPATH3, a cluster of genes over-expressed in CHA treatment mapped to pathways associated with energy metabolism, amino acid metabolism, carbohydrate metabolism, and metabolism of cofactors and vitamins (Figure S1). Altered nutrient metabolism in the rumen was expected due to the differences in the type of diets fed to the animals. Feeding a highly fermentable diet such as corn provides the energy needed by rumen microbes for several activities such as microbial protein synthesis [42]. In agreement with our study, Zhang et al. [43] reported ruminal increases in amino acids and sugars as well as increased concentrations of metabolites involved in aminoacyl-tRNA and amino acid biosynthesis pathways with increased level of corn-based concentrate fed to dairy cows. However, grain overload leads to rapid accumulation of volatile fatty acids and consequent low pH leading to dysbiotic rumen environment.

Functional analysis of the gene transcripts using KEGG cellular processing reference pathway level 3 showed that the number of genes mapped to the pathway associated with biofilm formation (*Escherichia coli*, *Vibrio cholera*, and *Pseudomonas aeruginosa*) was greater in CHA treatment (Figure S2). The formation of biofilms by microbial organisms is a form of survival strategy against stress and environmental change such as low pH [44] observed during SARA. The survival strategy of rumen microorganisms, especially the pathogenic bacteria, by biofilm formation poses health concerns because biofilm acts as a physical barrier against antimicrobial drugs and animal’s immune response [45]. One of the consequences of SARA is ruminal parakeratosis caused by accumulation of organic acids that enables the translocation of pathogens into the bloodstream causing systemic inflammation [46]. This study supports the concept that SARA is associated with ruminal pathogenesis. An increase in *E. coli* was positively associated with the severity of SARA symptoms [32]. Similarly, Khafipour et al. [47] reported increased virulence and adhesion factors in *E. coli* isolated during grain-induced SARA. Increased biofilm formation may also explain the high prevalence of antibiotic-resistant pathogens observed in grain-fed cattle that had not previously been administered antimicrobial agents [48]. In addition, these results may partly contribute to the reason why resistance to antimicrobial agents in *E. coli* and other pathogens increases with declining pH [49]. Further studies are needed to investigate the biological significance of these results to increase our understanding of pathogenesis of SARA.

## 4. Conclusions

This study provides a snapshot of the functional gene expression and active metabolic pathways of rumen microbiota in response to SARA. Our results revealed that several commensal cellulolytic rumen bacteria including the *Fibrobacter succinogenes* and two *Ruminococcus* species were reduced by acidotic challenge. Genes mapped to pathways involving metabolism of vitamin and co-factor, amino acid, carbohydrate, and energy, and three unique pathways of biofilm formation were enriched in the rumen of steers exposed to corn-induced acidosis.

## Figures and Tables

**Table 1:** Ruminal fermentation variables in beef cattle during the acidosis challenge.

Item	Treatment ^1^		SE	*p*-Value
	CHA	CON		
Lactate, mM	0.95	0.89	0.03	0.14
Total VFA, mM	114	98.5	3.36	0.01
Acetate, mM	62.4	64.9	1.87	0.91
Propionate, mM	35.8	25.3	2.76	0.01

^1^ CHA = corn-induced acidotic challenge, CON = control (no challenge).

## Supplementary Materials

The following are available online at https://www.mdpi.com/2076-2615/9/5/232/s1, Table S1: Chemical composition of the basal diet, Table S2: Abundance of unique KEGG genes, Table S3: Number of active KEGG genes per sample, Table S4: Abundance of transcriptionally active taxa, Table S5: Differentially enriched functional genes, Figure S1: KEGG mapping of functional genes overexpressed in steers challenged with sub-acute acidosis, Figure S2: Abundance of functional genes mapped to pathways associated with biofilm formation.
