# Supplementary material for: Metatranscriptomic Analysis of Sub-Acute Ruminal Acidosis in Beef Cattle
Source: Animals (Basel). 2019 May 12;9(5):232. doi: 10.3390/ani9050232 (PMC6562385; doi:10.3390/ani9050232)
Supplement: Supplementary file 1 [file animals-09-00232-s001.zip › Figure S1.docx]

Figure S1: KEGG mapping of functional genes overexpressed in steers challenged with sub-acute acidosis
