# Supplementary material for: Metatranscriptomic Analysis of Sub-Acute Ruminal Acidosis in Beef Cattle
Source: Animals (Basel). 2019 May 12;9(5):232. doi: 10.3390/ani9050232 (PMC6562385; doi:10.3390/ani9050232)

Figure S2: Abundance of functional genes mapped to pathways associated with biofilm formation in beef cattle during acidosis challenge. Each bar represents each sample. CHA = corn-induced acidotic challenge, CON = control (no challenge).


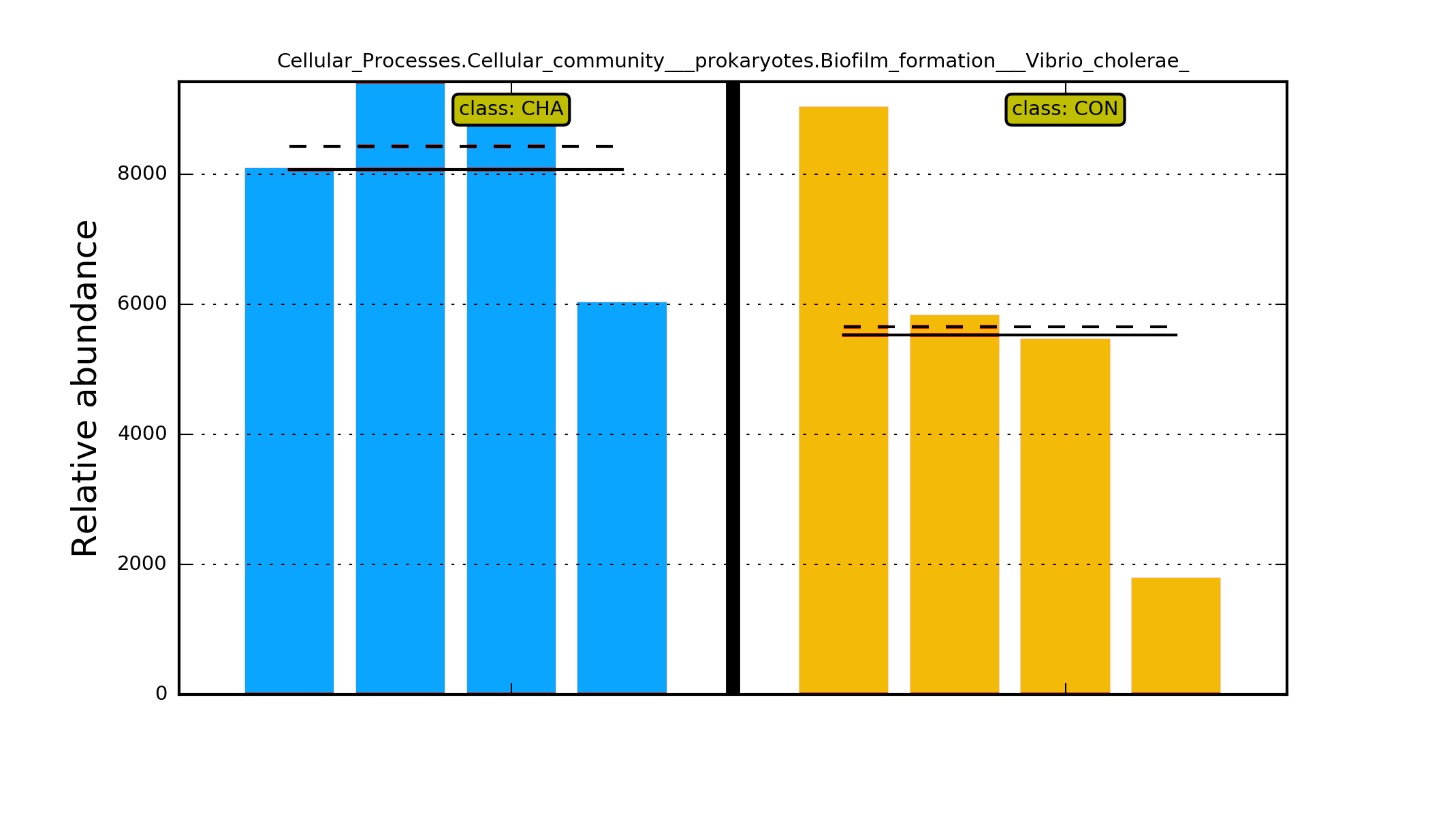


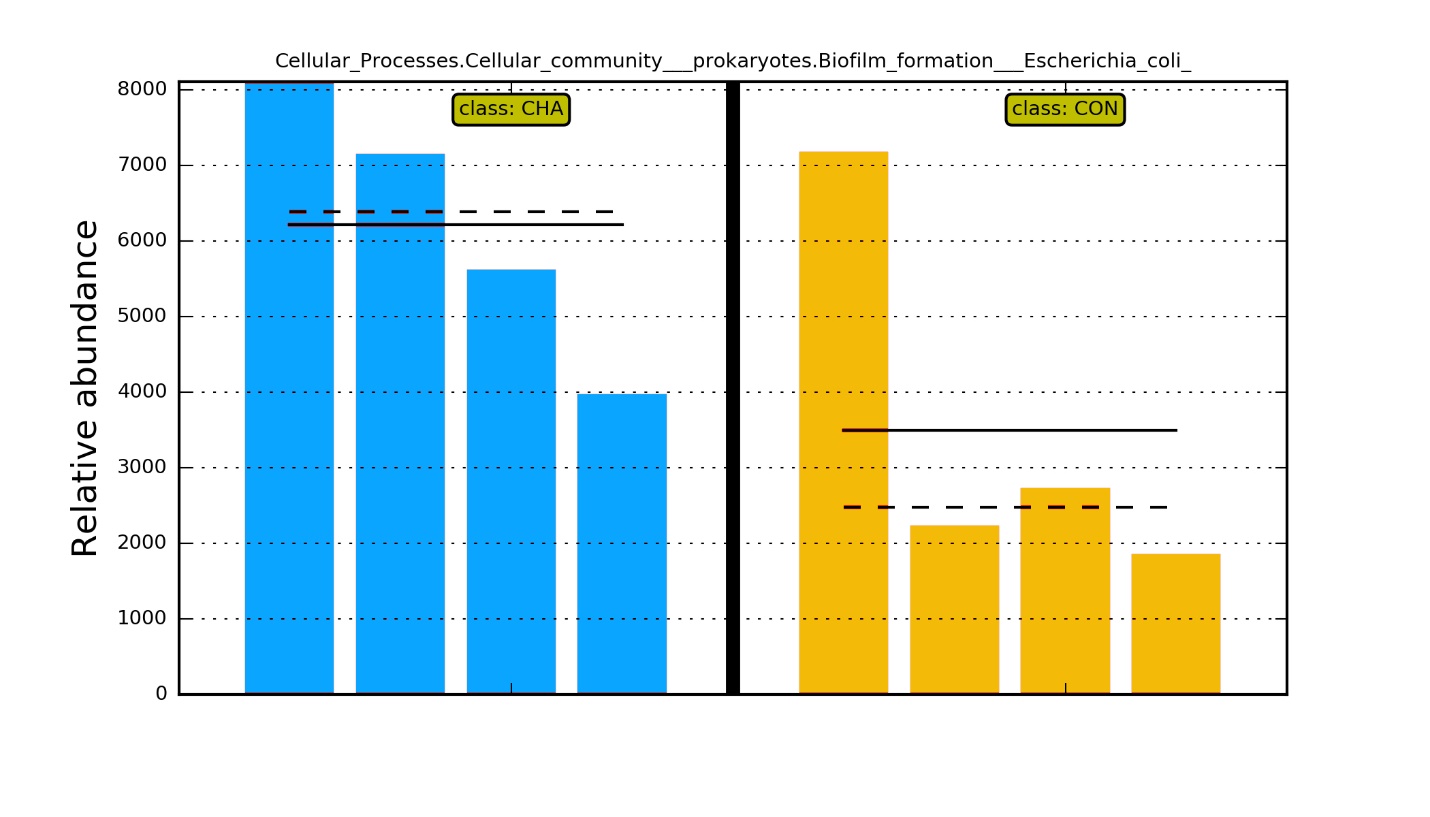


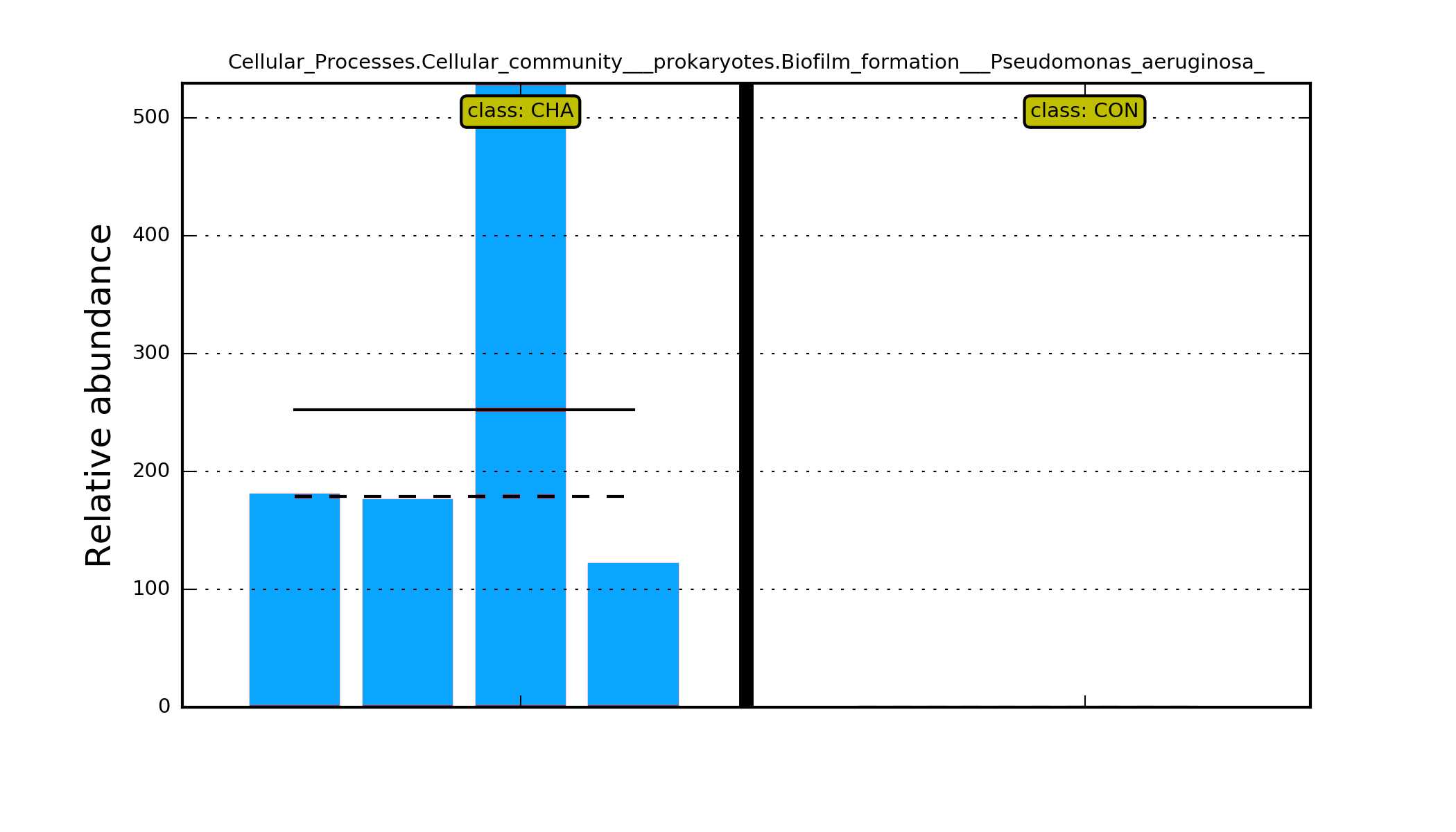

Supplement: Supplementary file 1 [file animals-09-00232-s001.zip › Figure S2.docx]
