# Supplementary material for: Metatranscriptomic Analysis of Sub-Acute Ruminal Acidosis in Beef Cattle
Source: Animals (Basel). 2019 May 12;9(5):232. doi: 10.3390/ani9050232 (PMC6562385; doi:10.3390/ani9050232)
Supplement: Supplementary file 1 [file animals-09-00232-s001.zip › Table S1.docx]

Table S1. Nutritional composition of the diet

|  | Red clover/orchard grass hay mixture | Concentrate supplement |
| --- | --- | --- |
| Dry matter (%) | 92.6 | 89.3 |
| NDF (% DM) | 58.9 | 45.3 |
| ADF (% DM) | 40.2 | 24.4 |
| Crude protein (% DM) | 11.4 | 14.3 |
| Ether extract (% DM) | NA^1^ | 2.44 |
| Starch (% DM) | NA | 23.6 |

NDF; neutral detergent fiber, ADF; acid detergent fiber

Concentrate contains corn gluten meal, soyhull, and cracked corn in equal proportions.

Guaranteed analysis of the mineral mix (Hubbard feeds, Mankato, MN); 8.0% calcium, 6% phosphorus, 14% magnesium, 12 ppm cobalt, 2000 ppm copper, 55 ppm iodine, 4800 ppm manganese, 36.4 ppm selenium, 4800 ppm zinc, 100,000 IU/lb vitamin A, 20,000 IU/lb vitamin D, and 250 IU/lb vitamin E.

^1^Not measured
